# The role of lung biopsy for diagnosis and prognosis of interstitial lung disease in systemic sclerosis: a systematic literature review

**DOI:** 10.1186/s12931-024-02725-1

**Published:** 2024-03-23

**Authors:** A Damiani, M Orlandi, C Bruni, G Bandini, G Lepri, C Scaletti, C Ravaglia, F Frassanito, S Guiducci, A Moggi-Pignone, M Matucci-Cerinic, V Poletti, L Tofani, TV Colby, S Bellando Randone, Sara Tomassetti

**Affiliations:** 1 Department of Clinical and Experimental Medicine, Rheumatology Unit, University of Florence, Careggi University Hospital, Florence, Italy; 2 Department of Medical and Surgical for Children and Adults, Modena, Italy; 3 Department of Experimental and Clinical Medicine, Division of Internal Medicine, University of Florence, Careggi University Hospital, Florence, Italy; 4 Pulmonary Unit, Department of Thoracic Diseases, Azienda USL Romagna, GB Morgagni-L-Pierantoni Hospital, Bologna University, Forlì, Italy; 5 Unit of Immunology, Rheumatology, Allergy and Rare diseases (UnIRAR), IRCCS San Raffaele Hospital, Milan, Italy; 6 Department of Statistics, Informatics and Applications, University of Florence, Florence, Italy; 7 Department of Pathology and Laboratory Medicine (Emeritus), Mayo Clinic, Scottsdale, AZ 13400 USA; 8 Department of Clinical and Experimental Medicine, University of Florence and Interventional Pulmonology Unit, Careggi University Hospital, Largo Brambilla 3, Florence, 50134 Italy

**Keywords:** Systemic sclerosis, Interstitial lung disease, Lung biopsy, Cryobiopsy

## Abstract

**Background:**

The prognostic and theragnostic role of histopathological subsets in systemic sclerosis interstitial lung disease (SSc-ILD) have been largely neglected due to the paucity of treatment options and the risks associated with surgical lung biopsy. The novel drugs for the treatment of ILDs and the availability of transbronchial cryobiopsy provide a new clinical scenario making lung biopsy more feasible and a pivotal guide for treatment. The aim of our study was to investigate the usefulness of lung biopsy in SSc ILD with a systematic literature review (SLR).

**Methods:**

PubMed, Embase and Cochrane databases were searched up to June 30, 2023. Search terms included both database-specific controlled vocabulary terms and free-text terms relating to lung biopsy and SSc-ILD diagnostic and prognosis. The SLR was conducted according to the Preferred Reporting Items for Systematic Reviews and Meta-analysis (PRISMA). Studies were selected according to the PEO (population, exposure, and outcomes) framework and Quality assessment of diagnostic accuracy studies (QUADAS) were reported.

**Results:**

We selected 14 articles (comprising 364 SSc-ILD patients). The paucity and heterogeneity of the studies prevented a systematic analysis. Diffuse cutaneous SSc was present in 30–100% of cases. Female predominance was observed in all studies (ranging from 64 to 100%). Mean age ranged from 42 to 64 years. Mean FVC was 73.98 (+/-17.3), mean DLCO was 59.49 (+/-16.1). Anti-Scl70 antibodies positivity was detected in 33% of cases (range: 0-69.6). All patients underwent surgical lung biopsies, and multiple lobes were biopsied in a minority of studies (4/14). Poor HRCT-pathologic correlation was reported with HRCT-NSIP showing histopathologic UIP in up to 1/3 of cases. Limited data suggest that SSc-UIP patients may have a worse prognosis and response to immunosuppressive treatment compared to other histopathologic patterns.

**Conclusions:**

The data from this SLR clearly show the paucity and heterogeneity of the studies reporting lung biopsy in SSc ILD. Moreover, they highlight the need for further research to address whether the lung biopsy can be helpful to refine prognostic prediction and guide therapeutic choices.

**Supplementary Information:**

The online version contains supplementary material available at 10.1186/s12931-024-02725-1.

## Background

Systemic sclerosis (SSc) is a connective tissue disease characterized by microvascular alterations, excessive collagen deposition and autoimmune dysregulation, mainly affecting women (female:male ranging from 3–8:1) with a peak of disease is between 45 and 64 years [1–3]. Pulmonary involvement occurs in more than 80% of SSc patients. Interstitial lung disease (ILD) and pulmonary arterial hypertension (PAH) account for up to 60% of the SSc-associated mortality [4–6].

High resolution computed tomography (HRCT) is the imaging gold standard to confirm the presence of ILD in SSc and is considered a sensitive and reproducible method for quantifying the extent of ILD, although radiomics may be more sensitive than visual analysis to capture features indicating SSc-ILD severity on HRCT [7, 8]. However, the radiologic and pathologic correlations between HRCT and lung biopsy features of SSc-ILD remain poorly investigated. The most relevant radiologic pattern observed in SSc-ILD is non-specific interstitial pneumonia (NSIP), but recent studies suggest that among SSc-ILD with late stage disease usual interstitial pneumonia (UIP) is the predominant pattern (87% of cases) and detailed pathologic studies in the early phase of the disease are lacking [9–11].

In SSc-ILD patients, lung biopsy is mainly performed when there is a discrepancy between clinical manifestations and HRCT findings and when other diseases, that may complicate SSc clinical course (e.g. lymphomas, lung carcinoma), must be excluded [9]. In selected cases, lung biopsy in combination with BAL may rule out infections, aspiration, and drug toxicity. However, the prognostic role of histopathological subsets and the utility of lung biopsy in guiding the therapeutic strategy in SSc-ILD has been largely neglected [9, 12]. Also the influence of coexistent connective tissue diseases (CTDs) potentially influencing the HRCT and lung biopsy results has also not been properly explored to date. The clinical approach to SSc-ILD contrasts with that of idiopathic interstitial pneumonias (IIPs) where robust data have shown that lung biopsy findings are pivotal in the diagnosis and prediction of prognosis. In fact, histopathological UIP is associated with a worse survival in comparison with other pathological patterns of lung injury [13, 14]. This different diagnostic approach between IIPs and SSc-ILD is due mainly due to: (1) the lack of effective treatment of ILD in SSc, (resulting in the use of clinical features for diagnosis and treatment choices), and (2) the risks and costs associated with the surgical lung biopsy that have discouraged its use in SSc ILD. However, the development of novel drugs for the treatment of UIP/IPF and progressive fibrotic lung disease, and the availability of transbronchial cryobiopsy provide for a new scenario [6, 15]. Moreover, the availability of tissue from cryobiopsy may significantly improve the understanding of SSc-ILD and the consequent management of these patients [9, 15–17].

Therefore, we have investigated, through a systematic literature review (SLR) of the available literature, the role of lung biopsy in the management of SSc patients in order to assess its utility either for both clinical practice and research purposes.

## Methods

### Methodology and quality assessment

The SLR was conducted according to the Preferred Reporting Items for Systematic Reviews and Meta-analysis (PRISMA). Studies were selected according to the PEO (population, exposure, and outcomes) framework outlined in supplementary data S1 (Data S1). Quality assessment of diagnostic accuracy studies (QUADAS) for articles included in the systematic review is summarize in supplementary table S2 (Table S2).

### Literature search strategy

PubMed, Embase and Cochrane databases were queried for any relevant publications. Each database was searched from its inception date until June 30th, 2023. Search terms included both database-specific controlled vocabulary terms and free-text terms relating to lung biopsy and SSc-ILD diagnosis and prognosis. Pubmed and Cochrane were interrogated for “biopsy AND (scleroderma OR systemic sclerosis) AND interstitial lung disease AND (diagnosis OR diagnostic OR prognosis OR prognostic)”. Embase was questioned for ' biopsy:ti,ab AND (scleroderma:ti,ab OR ‘systemic sclerosis’:ti,ab) AND ‘interstitial lung disease’:ti,ab AND diagnos*:ti,ab OR (‘biopsy’/exp AND ‘scleroderma’/exp AND ‘interstitial lung disease’/exp AND (‘diagnosis’ OR ‘diagnostic procedure’)).

### Eligibility criteria

**Fig. 1:**
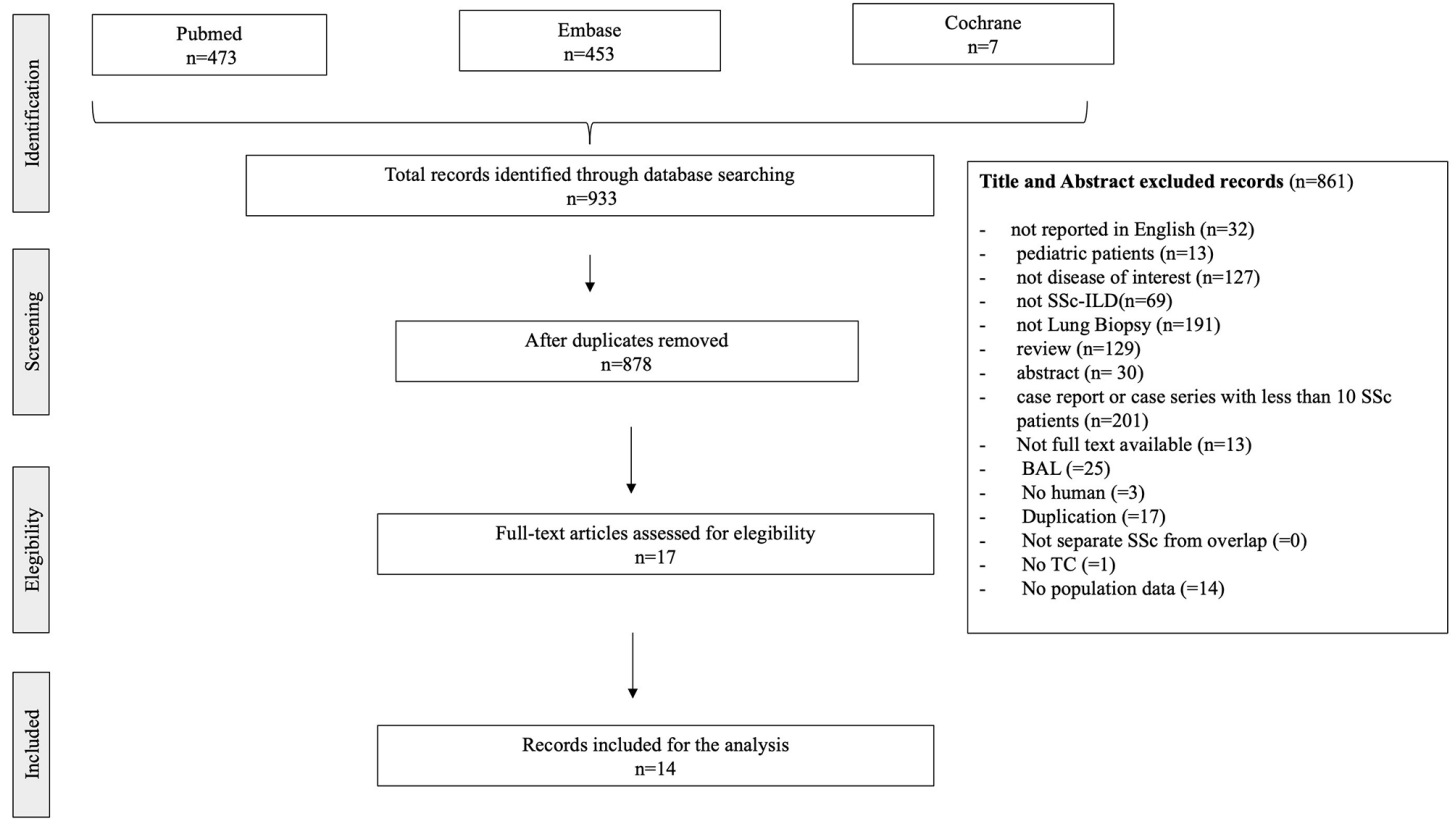
Flow-chart with details on the included and excluded papers Legend. BAL: Broncho-Alveolar Lavage; ILD: interstitial lung disease; SSc: Systemic Sclerosis

Articles published in English and addressing lung biopsy in adult patients with SSc-ILD were selected. Study inclusion criteria comprised peer-reviewed publication with population-based studies that reported an association between lung biopsy (all types) and SSc-ILD. Case reports, reviews, congress abstracts, letters to editor and editorials were excluded as well as case series with less than 10 SSc patients. Moreover, studies in which chest CT was not done were excluded. A detailed flow chart describing the study inclusion and exclusion process is available in Fig. 1.

### Data extraction

All identified articles were imported into Mendeley for screening. After deduplication, two screening rounds were performed, as described in Fig. 1. Firstly, two reviewers (A.D and F.F) separately evaluated titles and abstracts in terms of relevance for both biopsy and SSc-ILD. In the second round, full texts of the included articles were re-checked for eligibility. In case of disagreement during selection a third reviewer (S.BR) was consulted and a consensus reached.

The extracted contents were as follows: (1) The basic characteristics of the included studies: name of the first author, publication year, location of the studies, study design; study duration (months), total number of included patients, total number of SSc patients in the study, number of control population if present, SSc definition criteria (ACR 2013 criteria, ARA 1980 criteria); (2) other data: percentage of limited and diffuse cutaneous SSc, percentage of female patients, mean age (years), availability of PFTs, FVC (% pred) mean and SD, DLCO (% pred) mean and SD, prevalence of ILDs pattern by CT, biopsy technique (trans-bronchial, cryobiopsy, VATS biopsy, open surgical biopsy), prevalence of ILDs patterns on histopathology, availability and duration of follow up. Any biopsy findings on cellularity, cytokines and molecules expression data, previous or current treatments, autoantibodies, and procedure-related adverse events were also examined.

### Statistical analysis

Data for continuous variables are summarized as mean +/- standard deviation (median, minimum and maximum for not normally distributed variables); data for categorical variable are summarized as prevalence, with number and percentages. To compare mortality of UIP-SSc versus NSIP-SSc we pooled the Fisher’s study 5years survival, approximated using Kaplan-Meiers curve, with the Bouros’s study reported 5years survival [18, 19].

## Results

### Literature search results

The initial search retrieved 933 results from MEDLINE, 473 from Pubmed, 453 from Embase and 7 from Cochrane Collection through June 30, 2023. In Fig. 1, the flow-chart with details on the included and excluded papers are shown. After removal of duplicates, 878 papers were uploaded into Mendeley. First-stage screening by reviewing titles and abstracts excluded 861 publications for not addressing both lung biopsy and SSc-ILD; 17 articles were identified as relevant and assessed for eligibility. After the second-stage screening, 4 articles were excluded because they were not pertinent to the research item. To capture other potentially relevant articles, we also evaluated the full list of references from Reviews, which lead to manual inclusion of 1 additional article which was found to be pertinent to the research item. Finally, 14 articles met all the inclusion criteria constituting the final pool of this SLR (Fig. 1).

### Characteristics of the enrolled studies

Characteristics of the included studies are summarized in Table 1. The 14 studies selected in this systematic literature review included 364 SSc-ILD patients. The majority of studies (9/14) were retrospective single center studies. 11 studies based SSc diagnosis on ARA 1980 criteria, whereas two studies used the ACR 2013 diagnostic criteria [20, 21]. In 7 of the included studies, follow up data were available, with a mean duration of 5 years (+/- 4.44).

**Table 1:** Characteristics of the enrolled studies

Title	Author	Year	Journal	Study Design	Study duration months	N° of patients	Tot Number of SSc patients	N° of control	SSc definition	Diffuse cutaneous SSc %	female %	Mean age, years (sd)
Histopathologic Subsets of Fibrosing Alveolitis in Patients with Systemic Sclerosis and Their Relationship to Outcome	D. W. Bouros, A. U.: Nicholson, A. G.: Colby, et al.	2002	Am J Respir Crit Care Med	retrospective cohort	144	80	80	0	ARA 1980	30	NA	46 (11)
Suppressed signal transduction in the bronchial epithelium of patients with systemic sclerosis	M. A. Sehlstedt, G. N. Nilsson, K. Blomberg, A. et al.	2009	Respir Med	retrospective cohort	NA	40	23	17	ARA 1980	75	78	64 (10)
Arterial and interstitial remodelling processes in non-specific interstitial pneumonia: systemic sclerosis versus idiopathic	E. F. P. de Carvalho, E. R. de Souza, R. A’B Saber, et al.	2008	Histopathology	retrospective cohort	324	40	18	22	ARA 1980	78	100	45,82 (9,04)
Idiopathic and collagen vascular disease nonspecific interstitial pneumonia: Clinical significance of remodeling process	C. H. C. P. FelÌcio, E. R. Capelozzi, V. L.	2007	Lung	retrospective cohort	288	41	10	20	NA	NA	80	49.7 (12,7)
Anti-fibrotic effects of pirfenidone by interference with the hedgehog signalling pathway in patients with systemic sclerosis-associated interstitial lung disease	H. Z. Xiao, G. F. Liao, X. P. Li, et al.	2018	Int J Rheum Dis	clinical trial	NA	35	25	10	ARA 1980	100	84	48,1 (12,8)
Association of Interferon- and transforming growth factor b-regulated genes and macrophage activation with systemic sclerosis-related progressive lung fibrosis	R. B. S.-B. Christmann, P. Stifano, G. Borges, C. L. et al.	2014	Arthritis Rheumatol	prospective cohort	60	25	21	4	ARA 1980	52	100	44 (9,4)
Clinical Features of Idiopathic Interstitial Pneumonia with Systemic Sclerosis-Related Autoantibody in Comparison with Interstitial Pneumonia with Systemic Sclerosis	H. H. Yamakawa, E. Kitamura, H. Yamanaka, et al.	2016	PLoS One	retrospective cohort	NA	72	25	32	ACR 2013 criteria	NA	NA	NA
Structural features of interstitial lung disease in systemic sclerosis	N. K. M. Harrison, (A) R. Corrin, (B) Soosay, G. et al.	1991	Am Rev Respir Dis	retrospective cohort	NA	56	34	22	ARA 1980	58.8	76.5	46 (3)
Parenchymal and vascular interactions in the pathogenesis of nonspecific interstitial pneumonia in systemic sclerosis and idiopathic interstitial pneumonia	E. P. Franco de Carvalho, E. R. de Souza, R. Muxfeldt A’b Saber et al.	2008	Respiration	retrospective cohort	NA	40	18	22	ARA 1980	78	100	45.82 (2.19)
Clinically significant interstitial lung disease in limited scleroderma: histopathology, clinical features, and survival	A. S. Fischer, J. J. Groshong, S. D. Cool et al.	2008	Chest	retrospective cohort	NA	22	22	0	ACR 2013 criteria	0,00	64	NA
A long-term prospective randomized controlled study of non-specific interstitial pneumonia (NSIP) treatment in scleroderma	D. S. B. Domiciano, E. Borges, C. T. Kairalla et al.	2011	Clin Rheumatol	prospective cohor	30	18	18	0	ARA 1980	55.6	100	43,94 (9,36)
Centrilobular fibrosis: an underrecognized pattern in systemic sclerosis	R. B. B. de Souza, C. T. Capelozzi, V. L. Parra et al.	2009	Respiration	prospective cohor	30	28	28	0	ARA 1980	52,00	100	43,56 (9,23)
Morphometric evaluation of nitric oxide synthase isoforms and their cytokine regulators predict pulmonary dysfunction and survival in systemic sclerosis	E. R. A. J. Parra, A. C. Silva, L. O. Souza, et al.	2013	Braz J Med Biol Res	prospective cohor	30	23	23	0	ARA 1980	52,65	100	44,61 (7,02)
The major histopathologic pattern of pulmonary fibrosis in scleroderma is Nonspecific Interstitial Pneumonia	D. S. Kim, B. Yoo, J.S. Lee, et al.	2002	Sarcoidosis Vasculitis and Diffuse Lund Diseases	retrospective cohort	NA	19	19	0	ARA 1980	NA	68	42,3 (11,3)

Abbreviations: ACR: American College of Rheumatology

### Population

#### Clinical characteristics and lung function

Diffuse cutaneous SSc was variably present in the cohorts, ranging from 30 to 100% of cases. Female predominance was observed in all studies, ranging from 64 to 100%. Mean age in the studies ranged from 42 years to 64 years. Pulmonary function tests (PFTs) were available in all studies except one by Felicio et al. [22] Mean FVC was 73.98 (+/-17.3), mean DLCO was 59.49 (+/-16.1). PFTs value from Yamakawa et al. were not included in the analysis because of lack of separate data for patients with SSc [23].

#### Autoantibodies pattern

Only 3 studies had ANA data available showing ANA positivity in 96%, 73% and 100% of evaluated patients, respectively, [24–26]. Eight studies [19, 22, 23, 27–30] included data on anti-SCL70 antibodies positivity (median percentage 33%, range 0-69.6), 4 of them also evaluated ACA positivity (median percentage: 6.55%, min 3, max 32.5) [19, 23, 24, 27], and only 3 mentioned anti-RNP positivity (median percentage: 20%, min to (?), max 28) [23, 24, 27].

#### Previous or current treatments

In the included studies, previous treatment was poorly characterized. All patients in De Carvalho et al. study [31] (*N* = 18) and 20/25 patients from Xiao et al. [24], were naïve to treatment. The only previous treatments mentioned were corticosteroids (CCS, 5 patients) and cyclophosphamide (CYC, 3 patients). Five out of 14 studies provided information on ongoing treatment at the time of biopsy and the most common drugs used were CYC and CCS [18, 22, 26, 28, 29].

#### Lung biopsy techniques

Lung biopsies were obtained by surgery in 13 studies: VATS in 6) [18, 23, 25, 26, 28, 31, 32] and open surgical biopsy in 7) [22, 24, 28–30, 33, 34]. In one study, endobronchial biopsy was performed to study the bronchial mucosa [27]. None of the studies used transbronchial forceps biopsy or cryobiopsy for lung tissue sampling.

Few studies provided information on guidelines for surgical biopsy. The majority of studies highlighted the importance of avoiding CT honeycombing areas, e one study specified only that two or three biopsy specimens per patient were sampled [31] and in four studies, samples were taken in more than one lobe.^28 33 26^ None of the studies mentioned adverse events related to the biopsy procedure.

### HRCT features

Radiologic findings are summarized in Table 2. CTs or HRCTs were performed in all studies, but in nine radiologic features were not reported. In the remaining five studies HRCT findings were available for 118 patients. NSIP features were reported in 89/118 (75.4%), and UIP in 14/118 (11.9%) patients. Only Yamakawa et al., reported PPFE in 4/72 (5%) cases; the same Authors reported 10/72 cases (13.8%) as unclassifiable ILD by HRCT [19, 22, 23, 26–28, 31, 34].

**Table 2:** SSc-ILD, radiologic and histopathologic features and correlations

Ref.	LBx cases/TOTAL SSc-ILD cases (%)	Lung biopsyNSIP	Lung biopsy: cellular NSIP	Lung biopsy: fibrosing-NSIP	Lung biopsy: UIP	Lung biopsy. Other	N of cases with HRCT data	HRCT: NSIP/UIP/other	HRCT concordant with histopathology NSIP/UIP	HRCT discordant (HRCT -> histopathology)
Fischer, Chest 2008	22/27 (82%)	14/22 (64%)	1/22(4%)	13/22(60%)	8/22(36%)	-	9	8/1/0	5/1	3 NSIP -> UIP
Kim, Sarcoidosis VDLD 2002	19/19 (100%)	13/19 (68%)	2/19 (10%)^1^	11/19 (58%)	5/19(26%)	1 end stage lung (hcc only)	18	15/3/0	13/3	2 NSIP -> UIP
De Souza, Respiration 2009	28/28 (100%)	19/28 (68%)^2^	-	-	1/28 (3%)	6 CLF; 1 PH; 1 RB-ILD	19	18^3^/1/0	18^3^/0	1 UIP -> NSIP^4^
Bouros, AJRCCM 2022	80/80 (100%)	62/80 (77.5%)	15/80 (19%)	47/80 (59%)	6/80 (7.5%)	6 ESL; 4 RB-ILD, 1 sarcoidosis, 1 OP	-	-	-	-
de Carvalho, Histopathology 2008	18/18 (100%)	18/18 (100%)	-	18/18 (100%)	-	-	-	-	-	-
Felicio, Lung 2007	10/10 (100%)	10/10 (100%)	-	10/10 (100%)	-	-	-	-	-	-
Christmann, Arthritis and Rheum 2014	21/21 (100%)	16/21 (76%)	-	-	-	5 CLF	-	-	-	-
Domiciano, Clin Rheum 2011	24/24 (100%)	18/24 (75%)	14/24 (58%)	4/24 (16%)		4 CLF; 1 PH; 1 RB-ILD	-	-	-	-
Yamakawa, PLoS One 2016	32/72 (44%)	20/32 (62%)	-	20/32 (62%)	1/32 (3%)	11 unclassifiable	72	48/10/14^5^	-	-
Parra, Brazilian J Med and Biol Res 2013	23/23 (100%)	23/23 (100%)	11/23 (48%)	12/23 (52%)	-	-	-	-	-	-
Harrison, AJRCCM 1991	34/34 (100%)	-	-	-	-	34/34^6^	-	-	-	-
Franco De Carvalho, Respiration 2008	18/18 (100%)	18/18 (100%)	-	-	-	-	-	-	-	-
Xiao, Rheum Dis 2018	25/25 (100%)	-	-	-	-	-	-	-	-	-

Abbreviations. ILD: interstitial lung disease. HRCT: high resolution computed tomography. ESL: end stage lung; hcc: honeycomb changes; CLF: centrilobular fibrosis; PH: pulmonary hypertension; RB-ILD: respiratory bronchiolitis interstitial lung disease; OP: organizing pneumonia; NSIP: non specific interstitial pneumonia; UIP: usual interstitial pneumonia

(1) All cases were mixed NSIP (cellular and fibrotic), no cases of pure cellular NSIP were reported in this study. (2) 16/19 NSIP cases showed coexistent centrilobular fibrosis. (3) all 18 NSIP cases showed pure ground glass on HRCT, except one with reticulations. (4) one case with HRCT reticulations was misread by the radiologist as UIP, pathology confirmed NSIP. (5) other: unclass *N* = 10; PPFE *N* = 4. (6) this study didn’t define histopthologic patterns but described all cases as mixed fibrosis and inflammation similar to cryptogenic fibrosing alveolitis, nowadays defined idiopathic pulmonary fibrosis

### Histopathology

#### Major histopathology findings

Major histopathology findings are summarized in Table 2. In 12 studies, the histopathologic patterns are described, but 4 of them selected only NSIP cases. In the remaining 8 studies (261 LBx) the prevalence of NSIP, detected at the biopsy, was 70.9% (185/261 lung biopsies). The other histopathologic features were UIP in 21/152 cases (13.8%); end stage lung in 7/152 cases (4.6%), centrilobular fibrosis in 15/152 cases (9.8%), RB-ILD in 6/152 (3.9%), and pulmonary hypertension in 2/152 cases (1%) [18, 22–24, 26, 27, 30, 33, 34]. Only one study reported 11/32 biopsies as unclassifiable [23].

In 5 studies, NSIP was further classified into cellular or fibrotic (Table 2). Across all studies, fibrotic NSIP was the most prevalent feature in 63.4% (range 16–100%) of cases, while cellular NSIP was less frequently reported: 16.1% (range 0 to 58%).

The presence of CLF (interstitial fibrosis centered on membranous and respiratory bronchioles associated to foreign matter in the lumen due to microaspiration) was assessed in two studies only [28, 35]. Christmann et al. evidenced the presence of CLF in 5/ 21 patients, whereas the other 16 patients presented a NSIP pattern [28]. De Souza et al. showed that CLF was present as an isolated finding in 21% of patients, while some focal regions of this pattern were also found in 84% of patients with predominant NSIP [35].

#### Radiologic-pathologic correlations

The 4 studies reporting radiologic-pathologic correlations in 73 patients are summarized in Table 2.

Harrison et al. (year of publication 1991) showed that SSc patients without apparent CT changes can have pathologic findings on biopsy: 8 upper or middle lobe biopsies from regions defined as normal by CT were found to have not otherwise specified fibrosis histologically. In 21 cases, CT and histology were concordant in detecting ILDs and 3 normal lungs. In all 3 cases negative both at CT and histology electron microscopy could detect early changes [33].

Three studies (with a total of 46 cases) reported the correlation between HRCT and pathologic patterns: 41 cases showed a NSIP-HRCT pattern, and among them in 36 cases NSIP-HRCT was concordant with histopathology (88%, range 62-100%). Five NSIP-HRCT cases were histologically classified as UIP, (12%) [19, 26, 35]. All UIP-HRCT cases had honeycombing and were confirmed by as UIP on biopsy (4/27 total cases, 15%) [19, 26]. De Souza et al. reported one case of radiological UIP classified as NSIP by pathology [35].

De Souza evaluated HRCT features of patients with CLF and described that centrilobular or airway centered and patchy distribution of the lung involvement were features of CLF patients, while patients with histological NSIP showed peripheral and more diffuse distribution of ground-glass at HRCT: CLF was always associated with coexisting esophageal dilation (> 4 cm diameter) and with a higher frequency of centrilobular nodules (83%) [35].

#### Cellular findings on histopathology

Biopsy cellularity was examined in a few studies, either on bronchial mucosal biopsies or lung samples. In 23 patients, Sehlstedt et al. evaluated endobronchial biopsies and reported a higher number of neutrophils [27]. In lung biopsies, Harrison et al. evaluated neutrophils, eosinophils, macrophages, lymphocytes, and lymphoid aggregates in o the alveolar spaces and interstitium (determining inflammation): plasma cells were found in the interstitium and lymphoid aggregates and germinal centers were adjacent to bronchioles [33]. In lung biopsies, Yamakawa et al. found plasma cells infiltrate, lymphoid aggregates, germinal centers and fibroblastic foci, both in patients with SSc-ILD and ILD patients with SSc antibodies but without other clinical features of SSc [23].

#### Molecular findings on histopathology

In 4 studies, data on gene expression, cytokines and other molecular findings in lung or endobronchial biopsies were reported [27]. In mucosal endobronchial biopsies, Sehlstedt et al. found a lower epithelial IL-8 and NFkB expression in SSc-ILD samples compared to controls [27]. Christmann et al. compared gene expression of macrophage markers, chemokines, collagen, as well as transforming growth factor β– and interferon (IFN)–regulated genes, in lung biopsies of SSc-NSIP and controls. They found that expression of these genes correlated with progressive lung fibrosis defined by progression in radiological score (FibMax). Immunohistochemistry confirmed increased markers of collagen (COL1A1), IFN (OAS1 and IFI44), and macrophages (CCL18 and CD163). Moreover, several genes correlated with both the change in FibMax and the change in % predicted forced vital capacity, including IFN and macrophage markers, chemokines, and heat-shock proteins [28]. Parra et al. evaluated different expression of proteins regulating NO synthesis, and found that higher levels of iNOS, IL-13 and bFGF expression in lung biopsies of SSc patients with cellular and fibrotic NSIP correlated with the amount of parenchymal fibrosis [30]. More recently Xiao et al. evaluated 25 lung biopsies in SSc-ILD and found that the hedgehog pathway activation was increased in the lung tissue of SSc-ILD patients and this was decreased by pirfenidone, Sufu (suppressor of fused) was upregulated in lung fibroblasts after pirfenidone challenge, and pirfenidone inhibited the phosphorylation of GSK-3b signalling [24].

### Prognostic significance of histopathology features

#### Mortality

Among the 14 selected studies, only 4 studies reported SSc-ILD mortality data [18, 19, 23, 29], and only 2 reported the histopathology correlation with survival [18, 19]. Major findings are summarized in Table 3.

**Table 3:** Prognostic significance of histopathology findings in SSc-ILD

Ref.		total	NSIP	UIP	other
Bouros, AJRCCM 2002	SSc cases with LBx	80	62	12^1^	6
	Treatments: CSSonly/CYC/AZA	13/26/12	-	-	-
	Deaths N (%) - FUP 74.5 (16–62)months	22 (30)	16 (26)	6 (50)	-
	5 years survival^2^	-	90%	82%	-
	10 years survival^2^	-	69%	29%	-
	median FVC change at 1 and 3 years	-1.7% and − 2.5%	-	-	-
	median DLco change at 1 and 3 years	-0.3% and − 1.8%^3^	-	-	-
Fischer, Chest 2008	SSc cases with LBx	22	14	8	-
	Treatments: CSS + CYC or CSS + AZA	unk	-	-	-
	Deaths N (%)	11 (50)	6 (43)	5 (63)	-
	**median survival time in years** ^**4**^	**-**	**15.3**	**3**	-
Kim, Sarcoidosis VDLD 2002	SSc cases with LBx	19	13	5	-
	Treatments: CSSonly/CSS + CYC	1/12	0/12	1/0	-
	> 15% FVC and DLco improvement after treatment, N	-	5	0	-
	CRP score: N improved/ N worsened	-	5/0	0/5	-
	CRP score after treatment	-	-11.8 (8.9)	1.5 (3.4)	-
De Souza, Respiration 2009	SSc cases with LBx	28	19	1	6 CLF (2 other^^)
	Treatment: CYC/antiGERD only		18/0	-	0/6
	Δ % FVC decline at 1 year, median (SD)		–2.15 (11.28)	-	–3.87 (6.63)
	Δ % FEV1 decline at 1 year, median (SD)		–1.61 (10.54)	-	–9.88 (12.14)
	Δ % DLco decline at 1 year, median (SD)		–11.63 (20.98)	-	-17.06 (45.59)
Domiciano, Clir Rheum 2011	SSc cases with LBx	18	18^5^		-
	Treatments: CYC/CYC + PRED	9/9	9/9	-	-
	Deaths in 2 years	2 (11%)	2 (11%)		-
Yamakawa, PlosOne 2016	SSc cases with LBx	32	20	1	11 unclass
	Deaths N (%) - FUP 2.84 (0.15–17.25)years	18 (20)			
	5 years mortality	24.4%			

Abbreviations: NSIP: non specific interstitial pneumonia; UIP: usual interstitial pneumonia. LBx: lung biopsy. SSc: systemic sclerosis. CSS: corticosteroids. AZA: azathioprine. CYC: cyclophosphamide. FUP: follow-up. GERD: gastroesophageal reflux. DLco: carbon monoxide diffusion; FVC: forced vital capacity

Notes: (1) 6 UIP and 6 End Stage Lung; (2) survival difference between NSIP and UIP/ESL not statistically significant *p* = 0.33; (3) only DLco decline at 3 years was significantly linked to worse prognosis at multivariable analysis *p* = 0.003; (4) statistically significant *p* = 0.007; (5) 9 cellular NSIP and 9 fibrotic NSIP

Compared to UIP, NSIP showed better survival rates, but only in the Fisher et al. study did this reach statistical significance (median survival in years 15.3 for NSIP compared to 3 for UIP, *p* = 0.007) [18, 19]. Pooling the 5 years mortality from the two studies, the difference between histopathologic UIP-SSc and NSIP-SSc didn’t reach statistical significance: overall mortality for UIP-SSc was 37.5% (6/16) compared to 13.8% (9/65), *p* = 0.06 [18, 19]. Bouros et al. reported no difference in survival for UIP vs. NSIP nor for PFTs trends between fibrotic and cellular NSIP (15 and 47 total cases respectively) [18]. Baseline FVC and DLco, higher BAL eosinophils, DLco deterioration at 3 years and honeycombing at CT significantly correlated with mortality of SSc-ILD [18, 23].

Two prognostic studies compared CTD-ILD to idiopathic NSIP (iNSIP) and there was no mortality difference [22, 31]. Felicio et al. compared 20 iNSIP to 21 CTD-NSIP (10 were SSc-NSIP) confirming the overall good prognosis of NSIP in both groups (overall survival 135 months for iNSIP and 227 months for CTD-NSIP) [22]. De Carvalho et al. compared 22 iNSIP to 18 SSc-NSIP (all fibrotic) finding a higher collagen and elastic fibers content in the SSc-NSIP group, but without prognostic differences on univariate Kaplan Meyer analysis [31].

#### Disease progression

In 3 studies the functional decline of SSc-ILD was evaluated (Table 3) [18, 26, 35]. Only Kim et al. compared the functional decline between NSIP and UIP, reporting a better outcome after immunosuppressive treatment for NSIP compared to UIP (15% FVC improvement in 5/12 NSIP cases treated with CCS and CYC, compared to no improvement in the 5 UIP patients and a decrease in the CRP score after treatment only in the NSIP group) [26]. Only De Souza compared functional decline between NSIP and CLE without finding any statistically significant difference [35].

## Discussion

This systematic review on the utility of lung biopsy in SSc ILD clearly highlights the need for further research on this topic. Over the last 20 years, only a limited number of studies have employed lung biopsy either in the evaluation and management of patients with SSc-ILD or in order to better understand the pathophysiology of SSc. The present review highlightss the paucity and heterogeneity of the studies in terms of population selection, study aims and data collection. These limitations do not permit a systematic analysis and no solid conclusions on the role of biopsy in SSc-ILD diagnosis and management can be drawn.

Acknowledging those limitations, the most interesting finding of this SLR is that definition of histopathological pattern in SSc-ILD may have a prognostic and therapeutic significance. Only the Fisher study was sufficiently powered to detect a statistically significant difference in mortality among subgroups, providing very low quality evidence that in SSc-ILD UIP pattern may correlate with a higher mortality and a worse response to immunosuppressive treatment compared to NSIP [19, 26]. This finding is well established in IIPs and in rheumatoid arthritis related ILD (RA-ILD) [36]. The PANTHER trial has clearly shown that in UIP/IPF immunosuppression is detrimental and more recently the TRAIL-1 trial has shown that pirfenidone significantly slows lung function decline only in RA-ILD with UIP pattern but not with other patterns [37, 38]. Without further studies these findings cannot be extended to SSc-ILD where UIP pattern may have a different prognostic and theragnostic implications. The Park et al. study was excluded from the present SLR because of the absence of SSc-ILD population data [39]. However, it is important to underline that those Authors found a higher survival rate in CTD-ILD patients compared to IIPs, with CTD-UIP having a better prognosis compared to IPF-UIP, thus suggesting a possible different pathobiological background and prognosis for UIP related to SSc compared to its idiopathic counterpart [39]. In this systematic review, only one study compared the prognosis of cellular and fibrotic NSIP, but it failed to find a meaningful difference [18]. This is strikingly divergent from what is known in IIPs and in other ILDs, where a cellular NSIP has a better prognosis and treatment response compared to fibrotic NSIP [40]. The clinical significance of histopathologic findings in SSc-ILD may also vary over time and among patient subsets. SSc-ILD histopathologic features of the overall population at baseline may differ from those of progressive SSc-ILD. In progressive fibrotic SSc-ILD the prevalence and clinical impact of histopathologic UIP remain completely unexplored. Neither the SENSCIS, nor the INBUILD trial were designed according to histopathology data, therefore the real impact of molecular or morphological histopathological features on treatment response remains unexplored and lung biopsy role for SSc-ILD patients management continues to be neglected [41, 42]. The paucity of data reviewed in this SLR underline the urgent need to explore the universe of SSc-ILD patterns and pathobiological prognostic factors.

In SSc-ILD the UIP-HRCT pattern is rarely found, as it was seen in 13% of cases according to our review. In fact, this SLR confirmed that NSIP-HRCT is be the most prevalent pattern in SSc-related ILD (75%). However, there are limitations to consider. First of all, the majority of the studies predate the 2011 IPF guidelines that defined the diagnostic categories of the fibrotic patterns on HRCT. Moreover, several studies may have had selection bias including a priori only NSIP-SSc, excluding those studies the prevalence of NSIP-HRCT is 61.3%. The studies reviewed in this SLR show that HRCT-pathologic pattern correlation is imprecise. Histological UIP pattern may be present in up to one third of radiological fibrotic NSIP [19].

Among the other histopathologic patterns described in this review, CLF has been explored in 2 studies, with an overall prevalence of 9.8% in SSc-ILD. This pattern seems to correlate with chronic aspiration, a common complication of SSc, and does not seem, with very limited data, to have a meaningful prognostic impact. Given the paucity of studies and the high risk of bias no firm conclusion can be drawn. Moreover, PPFE and unclassifiable ILD, introduced 10 years ago in the histological classification [40], have been reported only in a few studies, and thus remain to be further explored [23]. Radiological PPFE was reported by Enomoto et al. to occur in 19% of patients with CTD-ILD (in 43% of patients with SSc-ILD), and PPFE-like HRCT lesions increased significantly the risk of death for respiratory causes (hazard ratio: 4.10, 95% confidence interval: 1.33–12.65, *p* = 0.01) [43]. In SSc, Bonifazi et al. have shown the important negative prognostic impact of radiological PPFE, but its biological mechanisms are to be further elucidated [23, 44].

The lung biopsy techniques reported in this SRL are obsolete and therefore we may advocate for future studies relying on more innovative and safer biopsy approaches such as transbronchial cryobiopsy [45]. The number of segments and lobes biopsied is incompletely reported and in many studies only one lobe was biopsied, introducing a risk of potential bias due to discordance of histopathologic features known to occur in different lobes [46]. Future studies should investigate optimal site(s) for lung biopsy using HRCT or more sophisticated guiding systems to allow precise radiologic-pathologic correlations [47]. Cryobiopsy performed in experienced centers for the diagnosis of ILDs is safe [45]. However, the safety data in SSc-ILD are limited. Both the lung function impairment and the possible vascular involvement should be carefully considered in SSc-ILD patients. There are no absolute contraindications to biopsy, but the indication for lung biopsy in patients with pulmonary hypertension or poor lung function should be very carefully balanced against the increased risk of possible severe complications.

Most of the included studies in this SLR were performed before the clinical use of antifibrotic therapies which now provide a much more extensive therapeutic armamentarium: different histopathologic patterns may predict different responses to drugs. In SSc-ILD, Xiao et al. showed the efficacy of pirfenidone as an anti-fibrotic compound in SSc fibrosis-related pathways [24]. In SSc patients, Parra et al. pointed out the importance of selectively inhibit inducible Nytric Oxyde Synthetasis pathway to avoid reperfusion damage during vasodilators treatments, suggesting the need of further study to understand the mechanisms of action of available drugs and to develop new therapies [30]. Novel transcriptomics technique are emerging and the importance of a lung tissue biobank is of critical importance for future development of targeted treatments [48]. There is an urgent unmet need to define histopathological findings in SSc-ILD. Unlike for the idiopathic ILDs, in SSc the real prevalence and significance of NSIP and UIP histopathological patterns remain largely unclear. Understanding whether these morphological patterns have therapeutic or prognostic significance is of major importance. molecular characterization of diseased lung may become part of the future landscape of precision treatment and may supercede morphologic classification and the UIP/NSIP dichotomy. In either case cryobiopsy remains a relatively safe biopsy alternative for traditional histopathologic evaluation and for tissue procurement for molecular studies.

In conclusion, the data obtained from this SLR suggest that lung biopsy does provide discriminating data characterizing pulmonary involvement in SSc patients. Future studies are needed to address whether it can be helpful to either refine our prognostic prediction or to guide treatment.

### Electronic supplementary material

Below is the link to the electronic supplementary material.

**Supplementary Material 1:** Supplementary data S1

**Supplementary Material 2:** Supplementary Table S2

## Data Availability

Dataset is available through request to the corresponding author.
