# Supplementary material for: The role of lung biopsy for diagnosis and prognosis of interstitial lung disease in systemic sclerosis: a systematic literature review
Source: Respir Res. 2024 Mar 23;25:138. doi: 10.1186/s12931-024-02725-1 (PMC10960984; doi:10.1186/s12931-024-02725-1)
Supplement: Supplementary file 1 — Supplementary Material 1: Supplementary data S1 [file 12931_2024_2725_MOESM1_ESM.docx]

**SUPPLEMENTARY DATA S1**

**Data S1. Patients exposure outcome (PEO) framework for eligibility of research question.**

*The****PEO*** *question format is used for qualitative research questions. Questions based on this format identify three concepts:*[*(1)****P****opulation, (2)****E****xposure, and (3)****O****utcome(s)*](http://my.ucs.ac.uk/Library/Subject-Guides/Nursing,-Midwifery--ODP/PICO-Searching2.pdf).

Questions are outlined below:

- In Systemic Sclerosis (SSc) patients (P), which is the prevalence (O) of BAL execution (E)?
- In Systemic Sclerosis (SSc) patients with secondary interstitial lung disease (ILD) (P), which is the prevalence (O) of BAL execution (E)?
- Which diagnostic criteria (O) were used in SSc-ILD patients (P), to define the presence of alveolitis at BAL (E)?
- In SSc-ILD patients (P), which is the prevalence (O) of alveolitis features at BAL (E)?
- In SSc-ILD patients (P), the presence of positive BAL (E) determines lung functional test alteration (O)?
- In SSc-ILD patients (P), the presence of positive BAL(E) determines FVC or FEV1 decrease (O)?
- In SSc-ILD patients (P), the presence of positive BAL (E) determines DLCO decrease (O)?
- In SSc-ILD patients (P), the presence of positive BAL (E) is associated to previous immunosuppressive treatment (O)?
- In SSc-ILD patients (P), the presence of positive BAL (E) determines worsening on prognosis (O)?
- In SSc-ILD patients (P), the presence of positive BAL (E) determines radiological progression of lesions in follow up (O)?
- In SSc-ILD patients (P), the presence of positive BAL (E) determines clinical worsening (O)?
- In SSc-ILD patients (P), the presence of positive BAL (E) determines progressive functional deterioration (O)?
- In SSc-ILD patients (P), the presence of positive BAL (E) determines death for respiratory failure (O)?
- In SSc-ILD patients (P), the presence of positive BAL (E) is treated with specific therapy (O)?
- In SSc-ILD patients (P), the presence of positive BAL (E) is treated with immunosuppressive therapy (O)?
