# Supplementary material for: The role of lung biopsy for diagnosis and prognosis of interstitial lung disease in systemic sclerosis: a systematic literature review
Source: Respir Res. 2024 Mar 23;25:138. doi: 10.1186/s12931-024-02725-1 (PMC10960984; doi:10.1186/s12931-024-02725-1)
Supplement: Supplementary file 2 — Supplementary Material 2: Supplementary Table S2 [file 12931_2024_2725_MOESM2_ESM.doc]

**SUPPLEMENTARY** **TABLE S2**

**Table S2. Quality assessment of diagnostic accuracy studies (QUADAS) for articles included in the systematic review.**

|  | bias patient selection | bias index test | bias reference standard | bias flow & timing | applicability patient selection | applicability index test | applicability reference standard |
| --- | --- | --- | --- | --- | --- | --- | --- |
| Bouros et al (19) | 0 | 0 | 0 | 0 | 0 | 0 | 0 |
| Cailes et al (20) | 0 | 0 | 2 | 0 | 0 | 0 | 0 |
| Clements et al (21) | 0 | 0 | 0 | 0 | 0 | 0 | 0 |
| De Santis et al (22) | 0 | 0 | 0 | 0 | 0 | 0 | 0 |
| Goh et al (23) | 2 | 0 | 0 | 0 | 0 | 2 | 0 |
| Goldin et al (24) | 0 | 0 | 0 | 0 | 0 | 0 | 0 |
| Hant et al (25) | 0 | 0 | 0 | 0 | 0 | 0 | 0 |
| Kowal-Bielecka et al (26) | 0 | 0 | 0 | 0 | 0 | 0 | 0 |
| Moodley et al (27) | 0 | 0 | 0 | 0 | 0 | 0 | 0 |
| Nagasawa et al (28) | 0 | 0 | 0 | 0 | 0 | 0 | 0 |
| Prasse et al (29) |  | 0 | 0 | 0 | 0 | 0 | 0 |
| Salaffi et al (30) |  | 0 | 0 | 0 | 0 | 0 | 0 |
| Schmidt et al (31) | 0 | 0 | 0 | 0 | 0 | 0 | 0 |
| Southcott et al (32) | 0 | 0 | 0 | 0 | 0 | 0 | 0 |
| Volpinari et al (33) | 2 | 0 | 0 | 0 | 0 | 0 | 0 |
| Yilmaz et al (34) | 2 | 0 | 0 | 0 | 2 | 0 | 0 |
| Wells et al (35) | 0 | 0 | 0 | 0 | 0 | 0 | 0 |
| Behr et al (36) | 0 | 0 | 0 | 0 | 0 | 0 | 0 |

*QUADAS-2 comprises 4 domains: patient selection, index test, reference standard, and flow and timing. Each domain is assessed in terms of risk of bias; and the patient selection, index test, and reference standard domains are also assessed regarding applicability. 0= low risk; 1= high risk; 2= unclear risk.*
